# Noncompetitive Inhibition of 5-HT_3_ Receptors by Citral, Linalool, and Eucalyptol Revealed by Nonlinear Mixed-Effects Modeling

**DOI:** 10.1124/jpet.115.230011

**Published:** 2016-03

**Authors:** Gavin E. Jarvis, Roseli Barbosa, Andrew J. Thompson

**Affiliations:** Department of Physiology, Development and Neuroscience, University of Cambridge, Cambridge, United Kingdom (G.E.J.); Mestrado em Bioprospecção Molecular, Universidade Regional do Cariri, Crato, Brazil (R.B.); and Department of Pharmacology, Cambridge, United Kingdom (A.J.T.)

## Abstract

Citral, eucalyptol, and linalool are widely used as flavorings, fragrances, and cosmetics. Here, we examined their effects on electrophysiological and binding properties of human 5-HT_3_ receptors expressed in *Xenopus* oocytes and human embryonic kidney 293 cells, respectively. Data were analyzed using nonlinear mixed-effects modeling to account for random variance in the peak current response between oocytes. The oils caused an insurmountable inhibition of 5‐HT–evoked currents (citral IC_50_ = 120 *µ*M; eucalyptol = 258 *µ*M; linalool = 141 *µ*M) and did not compete with fluorescently labeled granisetron, suggesting a noncompetitive mechanism of action. Inhibition was not use‐dependent but required a 30-second preapplication. Compound washout caused a slow (∼180 seconds) but complete recovery. Coapplication of the oils with bilobalide or diltiazem indicated they did not bind at the same locations as these channel blockers. Homology modeling and ligand docking predicted binding to a transmembrane cavity at the interface of adjacent subunits. Liquid chromatography coupled to mass spectrometry showed that an essential oil extracted from *Lippia alba* contained 75.9% citral. This inhibited expressed 5‐HT_3_ receptors (IC_50_ = 45 *µ*g ml^−1^) and smooth muscle contractions in rat trachea (IC_50_ = 200 *µ*g ml^−1^) and guinea pig ileum (IC_50_ = 20 *µ*g ml^−1^), providing a possible mechanistic explanation for why this oil has been used to treat gastrointestinal and respiratory ailments. These results demonstrate that citral, eucalyptol, and linalool inhibit 5-HT_3_ receptors, and their binding to a conserved cavity suggests a valuable target for novel allosteric modulators.

## Introduction

**Fig. 1.**
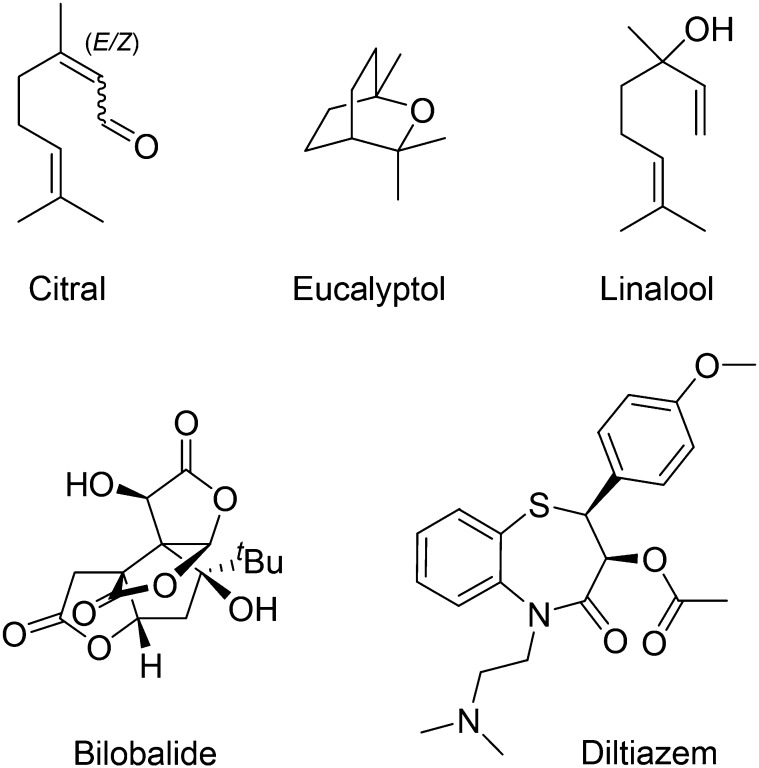
Chemical structures of diltiazem and the terpenoids used in this study.

The natural oils citral, eucalyptol, and linalool (Fig. 1) are widely used as scents and flavorings in pharmaceuticals, foods, and health care products. Related compounds are reported to have antioxidant, anti-inflammatory, antiproliferative, antimicrobial, and acaricide activities. They belong to the terpenoid class of molecules that contains both structurally complex (e.g., bilobalide and ginkgolide) and simple (e.g., thymol and menthol) compounds (Caputi and Aprea, 2011). Terpenoids have effects on a broad selection of both voltage-gated and ligand-gated ion channels, and some are noncompetitive ligands of 5-hydroxytryptamine 3 (5-HT_3_) receptors (Hall et al., 2004; Ashoor et al., 2013; Kessler et al., 2014; Lansdell et al., 2015; Ziemba et al., 2015).

5-HT_3_ receptors belong to the Cys-loop family of transmembrane ligand-gated ion channels, which are responsible for fast excitatory and inhibitory neurotransmission in the central and peripheral nervous systems. It includes vertebrate nicotinic acetylcholine, GABA and glycine receptors, invertebrate receptors such as the glutamate-gated chloride channel (GluCl) and resistance-to-dieldrin channels (RDL), and prokaryotic homologs such as *Erwinia* chrysanthemi ligand-gated ion channel (ELIC) and *Gloeobacter* violaceus ligand-gated ion channel (GLIC) (Thompson et al., 2010). Each channel comprises five subunits surrounding a central ion-conducting pore, and each subunit has three distinct domains referred to as extracellular, transmembrane, and intracellular. The orthosteric binding site (that occupied by endogenous agonist) is located in the extracellular domain at the interface of two adjacent subunits, where binding is coordinated by the convergence of six peptide loops (Hassaine et al., 2014). The transmembrane domain of each subunit contains four *α*-helices (M1–M4), with M2 from each forming a central ion-conducting pore; to aid comparisons of the channel-lining residues of M2 from different Cys-loop receptors, a prime notation is often used to define residue positions, with 0' representing a conserved charged residue at the cytoplasmic side of the membrane. The intracellular domain regulates receptor trafficking, intracellular modulation, and ion channel conductance, but remains structurally unresolved (Hassaine et al., 2014).

5-HT_3_ receptor ligands typically target extracellular and transmembrane domains. Competitive antagonists such as granisetron and tropisetron are used in the clinic to alleviate nausea and vomiting associated with chemotherapy, radiotherapy, and general anesthesia. There has also been limited use of partial agonists and competitive antagonists in the management of irritable bowel syndrome (Moore et al., 2013). Allosteric ligands include the anthelmintic ivermectin and general anesthetic propofol, both of which bind to cavities in the transmembrane domain of vertebrate, invertebrate, and prokaryotic channels (Nury et al., 2011; Chiara et al., 2013; Jayakar et al., 2013; Yip et al., 2013). Smaller terpenoids such as menthol and thymol also bind in transmembrane cavities, whereas more complex terpenoids such as bilobalide and ginkgolide bind in the channel of the 5-HT_3_ receptor and other Cys-loop receptors (Hawthorne et al., 2006; Thompson et al., 2010; Lynagh and Lynch, 2012; Lynagh and Laube, 2014).

Medicinal products containing terpenoids are widely available for the relief of gastrointestinal and respiratory disorders. Although the pharmacological mechanisms of their active ingredients are not well known, evidence suggests the actions of some may result from effects at 5-HT_3_ receptors. For example, menthol has an IC_50_ of 163 *µ*M at 5-HT_3_ receptors and blocks gut contractions at the same concentration (Heimes et al., 2011; Ashoor et al., 2013). The ginger extracts 6-gingerol and 6-shogaol similarly inhibit 5-HT_3_ receptors and 5-HT–mediated gut contractions with micromolar potency (Abdel-Aziz et al., 2006; Walstab et al., 2013, 2014). With an increasing number of reports also describing the effects of terpenoids at other receptors, the potential physiologic effects of these compounds deserve investigation (Oz et al., 2015).

In this study, we investigate the pharmacology of the terpenoids citral, linalool, and eucalyptol. We use nonlinear mixed-effects modeling to determine their effects on the electrophysiology of 5-HT_3_ receptors and, combined with flow cytometry and in silico docking, investigate their modes of action and potential binding sites. Using liquid chromatography coupled to mass spectrometry (LC-MS), we also report the terpenoid content of an essential oil extracted from *Lippia alba* and determine its effects on the contraction of isolated ileum and trachea.

## Materials and Methods

### 

#### Materials.

Citral, eucalyptol, linalool (Fig. 1), and 5-hydroxytryptamine were from Sigma-Aldrich (St. Louis, MO). Human 5-HT3A (accession number: P46098) subunit cDNA was provided by J. Peters (University of Dundee, Dundee, UK). Essential oil of *L. alba* was purchased and a species voucher was deposited on Prisco Bezerra Herbarium (Federal University of Ceará, Ceará, Brazil) with the following number identification: EAC-08474. Essential oil extracts from *L. alba* (OELa) were analyzed by LC-MS at Parque de Desenvolvimento Tecnológico (Ceara, Brazil).

#### Oocyte Maintenance.

Oocytes from *Xenopus laevis* were purchased from EcoCyte Bioscience (Castrop-Rauxel, Germany) and stored at 16°C in ND96 (96 mM NaCl, 2 mM KCl, 1 mM MgCl_2_, 5 mM HEPES, pH 7.5).

#### Cell Culture.

Human embryonic kidney 293 (HEK293) cells were grown on 90-mm round tissue culture plates as monolayers in Dulbecco’s modified Eagle’s medium (DMEM)/F12 (Gibco, Life Technologies, Carlsbad, CA) supplemented with 10% fetal bovine serum (Sigma-Aldrich) at 37°C in a moist atmosphere containing 5% CO_2_.

#### Receptor Expression.

5-HT3A subunit cDNA was cloned into pGEMHE for oocyte expression. cRNA was transcribed in vitro from a linearized plasmid cDNA template using the mMessage mMachine Ultra T7 Transcription kit (Ambion, Austin, TX). Stage V and VI oocytes were injected with 50 nl of 100–500 ng *µ*l^−1^ cRNA (5–25 ng injected), and currents were recorded 1–4 days postinjection.

5-HT3A subunit cDNA was cloned into pcDNA3.1 for expression in HEK293 cells (ThermoFisher Scientific, Waltham, MA). Cells were transiently transfected with this cDNA using polyethyleneimine (25 kDa, linear, powder; Polysciences Inc., Eppelheim, Germany). Thirty microliters of polyethyleneimine (1 mg ml^−1^), 5 *μ*g of cDNA, and 1 ml of DMEM were incubated for 10 minutes at room temperature, added dropwise to a 90-mm plate of 70–80% confluent HEK293 cells, and incubated for 2–3 days before use.

#### Electrophysiology.

Using two-electrode voltage clamp, *Xenopus* oocytes were routinely clamped at −60 mV using an OC-725 amplifier (Warner Instruments, Hamden, CT), NI USB-6341 X Series DAQ Device (National Instruments, Berkshire, UK), and the Strathclyde Electrophysiology Software Package v4.7.3 (University of Strathclyde, Glasgow, UK). Microelectrodes were fabricated from borosilicate glass (GC120TF-10; Harvard Apparatus, Edenbridge, Kent, UK) using a two-stage horizontal pull (P-97; Sutter Instrument Company, Novato, CA) and filled with 3 M KCl. Pipette resistances ranged from 0.8 to 2.0 MΩ. Oocytes were placed in a perfusion chamber made from 2-mm-wide × 30-mm-long silicon tubing that was cut in half lengthways (total volume ∼0.1 ml), and were perfused with ND96 at a rate of 12 ml min^−1^. Drug application was via a simple gravity-fed system calibrated to run at the same rate. For inhibition measurements, antagonists were routinely applied for 1 minute before coapplication with 5-HT. A 3-minute wash was used between compound applications. Oils were dissolved in buffer containing 1% DMSO, freshly prepared each day, and constantly stirred during the experiments.

#### Flow Cytometry.

HEK293 cells expressing the 5-HT_3_ receptor were grown in monolayers and harvested from a 90-mm culture dish using 10 ml of Trypsin-EDTA (Sigma-Aldrich) for 10 minutes at 37°C. Digestion was terminated by the addition of 25 ml DMEM + 10% fetal bovine serum, and cells were pelleted at low speed for 2 minutes. The pellet was resuspended in 3 ml of phosphate-buffered saline (137 mM NaCl, 8.0 mM Na_2_HPO_4_, 2.7 mM KCl, 1.47 mM KH_2_PO_4_, pH 7.4), and cells were filtered through a cell strainer (BD Falcon, Franklin Lakes, NJ). Competition binding was measured by incubating HEK293 cells with different concentrations of nonlabeled ligands and 10 nM fluorescent granisetron (G-FL). After 10-minute incubation, cells were pelleted and rapidly washed in phosphate-buffered saline before being resuspended in the same buffer and analyzed on a BD Accuri C6 flow cytometer (Becton, Dickinson and Company, Franklin Lakes, NJ) at 488-nm excitation/530-nm emission. The geometric mean was measured at each concentration of test compound and fitted to eq. 1 (see the following section) using a least-squares method (GraphPad Prism v4; GraphPad Software, La Jolla, CA).

#### Nonlinear Mixed-Effects Modeling.

Inhibition of 5-HT–induced currents was analyzed using Wings for NONMEM (distributed under a GNU General Public License) and NONMEM 7.3.0 (Icon PLC, Dublin, Ireland). NONMEM is typically used for population pharmacokinetic/pharmacodynamic analyses and is ideally suited to simultaneously model fixed nonlinear effects (e.g., drug concentration-response relationships) and random effects (e.g., variance in maximal peak current). Nonlinear mixed-effects modeling of this sort cannot easily be done using more familiar statistical packages. Specifically, NONMEM allows the modeling of the relationship between drug and response, and importantly, the random between-oocyte variance, which is considerable. NONMEM enabled a single unified model to be created that included all electrophysiological agonist and antagonist data. This comprised 532 individual data points from 55 oocytes from the following experiments: 1) control 5-HT concentration-response data, 2) 5-HT concentration-response data in the presence of test compounds, and 3) concentration-inhibition data at fixed 5-HT (typically 1.7 *µ*M) concentrations. The ability to allow for between-oocyte variance enabled raw peak currents to be analyzed, rather than normalized values. Normalization can obscure relationships between parameters, resulting in inaccurate and imprecise estimates of drug effects. Differences in peak currents and agonist potency between different oocytes were modeled with population variances (*ω*^2^) associated with model parameters.

A structural model defined the relationship between the independent variables (i.e., agonist and antagonist concentrations) and the peak current (dependent variable). The agonist response was modeled using the four-parameter logistic equation:(1)
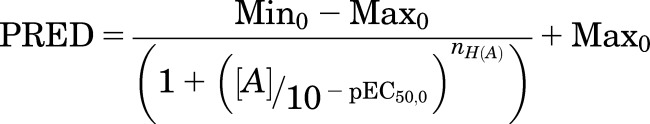
where PRED = predicted agonist-induced response, [*A*] = agonist concentration; Min_0_ = PRED when [*A*] = 0; Max_0_ = PRED when [*A*] = ∞; pEC_50,0_ = −log_10_[*A*] that induces PRED = (Max_0_ + Min_0_)/2; and *n_H(A)_* = agonist Hill coefficient. (In all cases, there was no basal current; hence, Min_0_ was fixed to zero.)

Current responses can change during an experiment. If this effect is not identified and quantified, it can distort estimates of agonist and compound effects. The effect of time on the agonist response was modeled linearly as:(2)
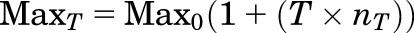
where Max_0_ = as in eq. 1; Max*_T_* = PRED when [*A*] = ∞ at time *T*; *T* = time (hours) from start of individual experiment; and *n_T_* = change in response per hour expressed as a proportion of the response at *T* = 0 (*n_T_* = 0 represents no change; *n_T_* = −0.1 represents a reduction of 10%, and *n_T_* = 0.1 represents an increase of 10% per hour).

The effect of the compounds on Max*_T_* was modeled as follows:(3)
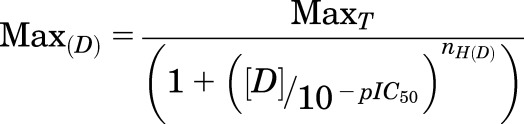
where Max*_T_* = PRED when [*A*] = ∞ in the absence of drug *D* at time *T* (eq. 2); Max_(_*_D_*_)_ = PRED when [*A*] = ∞ in the presence of drug *D* at time *T*; [*D*] = concentration of inhibitor drug; pIC_50_ = −log_10_[*D*] in the presence of which Max_(_*_D_*_)_ = Max*_T_*/2; and *n_H_*_(_*_D_*_)_ = inhibitor Hill coefficient. (This model assumes that, when [*D*] = ∞, PRED = 0.)

The effect of the compounds on agonist pEC_50_ was modeled as follows:(4)
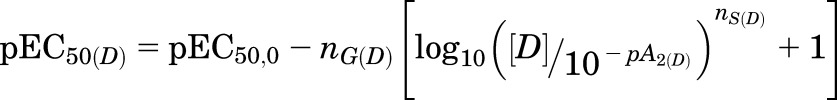
where: pEC_50,0_ = agonist pEC_50_ in the absence of drug *D* (eq. 1); pEC_50(_*_D_*_)_ = agonist pEC_50_ in the presence of drug *D*; [*D*] = concentration of drug; *pA*_2(_*_D_*_)_ = *pA*_2_ of drug *D*; *n_S_*_(_*_D_*_)_ = Schild coefficient for drug *D*; and *n_G_*_(_*_D_*_)_ = inhibition coefficient for drug *D*.

Variable Schild (*n_S_*) and inhibition (*n_G_*) coefficients enabled compound effects other than competitive-like inhibition to be modeled, and hypotheses about drug action on pEC_50_ to be statistically evaluated using likelihood ratio tests. For example, when *n_S_* = 1 and *n_G_* = 1, the effect on agonist pEC_50_ is consistent with that of a competitive antagonist, and when *n_G_* = 0, there is no effect of the drug on agonist pEC_50_.

Random effects (*η*) were included, enabling parameter values from different oocytes to differ from the typical, population parameter estimates. The relationship between population and individual oocyte parameters was modeled in the following two ways:(5)
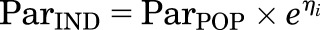
(6)
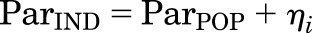
where Par_IND_ = individual parameter estimate; Par_POP_ = population parameter estimate; and *η_i_* = *η* value representing the difference between the population and an individual (*i*) parameter estimate. Values of *η* from all oocytes (*n* = 55) enabled a variance-covariance (OMEGA) matrix for random effects to be constructed. This allows the random variance inherent in different oocytes to be defined and correlations in estimated parameters to be identified.

Residual unexplained variance (RUV) defines the difference between the observed and predicted values for a given set of conditions. RUV was modeled as a function of PRED as follows:(7)
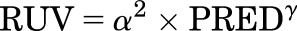
where RUV = residual unexplained variance between observed and predicted current; PRED = predicted current; *α*^2^ = variance parameter; and *γ* = variance scaling parameter. When *γ* = 0, RUV is constant irrespective of PRED and *α* = standard deviation of the residual variability; when *γ* = 1, RUV is proportional to the response (*α^2^* = RUV/PRED); when *γ* = 2, the coefficient of variation of the residual variability is constant (*α* = *SD*_RESID_/PRED).

Maximum likelihood was used to identify best fit parameters for specific models. A Laplacian method was used to obtain parameter estimates. The objective function used by NONMEM is the extended least squares. Parameter standard errors are from the covariance step in NONMEM. Models were evaluated by examining both population- and individual-weighted residuals and comparing Akaike and Bayesian information criteria. Specific hypothesis tests are defined in Table 1. They were performed by constraining parameters and comparing resultant differences in extended least squares values using likelihood ratio tests (Spalding and Jarvis, 2002; Mould and Upton, 2013).

**TABLE 1:** Output from the best fit model describing the effects of 5-HT and inhibitors on the peak current The best fit was selected based the stability of convergence on the objective function (extended least squares = 110.89) and a comparison of Akaike information criteria (1,132.64) and Bayesian information criteria (1,226.72) values to alternative models. The model was further evaluated by examining the relationship between observed and predicted values, and population and individual residuals.

Parameter	Population Parameter Estimate	Population Variance Model	Population SD(*ω*) or CORR*^b^*	Hypothesis Tests	P Value (LRT)	Interpretation
Agonist (5-HT)						
Min (*µ*A)	0 (fixed)	—	—	—	—	
Max (*µ*A)	4.94 ± 0.64	× EXP(ɳ_2_)	0.95 ± 0.10	H_0_: *ω*_2,2_=0	No result*^a^*	Max varies between oocytes
COV(*ɳ*_1_,*ɳ*_2_)			0.87 ± 0.05	H_0_: *ω*_1_,_2_=0	4 × 10^−9^	Max and pEC_50_ correlate strongly
pEC_50_	5.65 ± 0.03	+ *ɳ*_1_	0.18 ± 0.02	H_0_: *ω*_1,1_=0	4 × 10^−35^	pEC_50_ varies between oocytes
* n_H_*	2.94 ± 0.13			H_0_: *n_H(A)_*=3	0.64	Suggests three highly cooperative agonist binding sites
* n_T_*	−0.13 ± 0.06	+ *ɳ*_7_	0.19 ± 0.06	H_0_: *n_T_*=0	0.052	Weak evidence for a change in response with time
				H_0_: *ω*_7_=0	0.004	*n_T_* varies between oocytes
Citral						
pIC_50_	3.92 ± 0.05	+ *ɳ*_5_	0.16 ± 0.04	H_0_: *ω*_5_=0	2 × 10^−11^	pIC_50_ varies between oocytes
* n_H_*	1.34 ± 0.08			H_0_: *n_H_*=1	5 × 10^−6^	Indication of cooperative binding
* n_G_*	0 (fixed)			H_1_: {*n_G_,pA*_2_*,n_S_*}≠0	0.94	No evidence that citral changes agonist pEC_50_
Eucalyptol						
pIC_50_	3.59 ± 0.11	+ *ɳ*_4_	0.32 ± 0.07	H_0_: *ω*_4_=0	1 × 10^−16^	pIC_50_ varies between oocytes
* n_H_*	1.04 ± 0.11			H_0_: *n_H_*=1	0.76	Suggests one inhibitor binding site
* n_G_*	1 (fixed)			H_0_: *n_G_*=0	4 × 10^−15^	Strong evidence that eucalyptol changes agonist pEC_50_
pA_2_	3.09 ± 0.09	+ *ɳ*_3_	0.18 ± 0.06	H_0_: *ω*_3_=0	0.006	*pA*_2_ varies between oocytes
* n_S_*	1.70 ± 0.24			H_0_: *n_S_*=1	0.001	Schild slope greater than that for competitive antagonism
Linalool						
pIC_50_	3.85 ± 0.02	+ *ɳ*_6_	0.05 ± 0.02	H_0_: *ω*_6_=0	0.023	pIC_50_ varies between oocytes
* n_H_*	2.19 ± 0.26			H_0_: *n_H_*=2	0.46	Suggests two highly cooperative inhibitor binding sites
* n_G_*	0 (fixed)			H_1_: {*n_G_,pA*_2_*,n_S_*}≠0	0.11	No/weak evidence that linalool changes agonist pEC_50_
RUV model						
* γ*	1.30 ± 0.07			H_0_: *γ*=0	1 × 10^−63^	Residual error variance increases with the current amplitude
* α*	0.30 ± 0.01					

COV, covariance; LRT, likelihood ratio test.

^a^ No stable convergence was obtained without a random effect included.

^b^ Parameter estimates are shown ± standard errors.

#### Drug Effects at 5-HT_3_A_T6'S_ Receptors.

The effects of the compounds on 5-HT_3_A_T6'S_ receptors were also evaluated. A total of 113 data values from 11 oocytes comprising control 5-HT concentration-response and compound concentration-inhibition curves were incorporated into the wild-type receptor data set. Parameters were incorporated into the model that defined changes in pEC_50,_ pIC_50_, Max_0_, and *pA*_2_ values for 5-HT_3_A_T6'S_ receptors. These parameters were statistically evaluated as described earlier.

#### Dual Application.

Dual application studies were performed as previously described (Jarvis and Thompson, 2013). This is a simple method to determine whether two channel blockers share the same binding site (syntopic inhibition) or bind to separate locations (allotopic inhibition). For each test compound, inhibition of a supramaximal 5-HT–induced (100 *µ*M) response was measured alone and in combination with bilobalide (BB) and diltiazem (DTZ), both well characterized 5-HT_3_ blockers (Fig. 1; Thompson et al., 2011a). Concentrations of the test compounds, BB and DTZ, were selected such that they caused approximately 62% inhibition when used alone. From the results of these experiments, predicted levels of inhibition were derived for both allotopic and syntopic modes of action. These predictions were compared with data obtained experimentally using the same concentrations of the inhibitors acting together. For each oocyte, the change in the amplitude of the 5-HT response over time was monitored and taken into account. Inhibition by drugs was quantified in relation to interpolated control responses for each time point.

The dual application measurements were compared statistically with allotopic and syntopic predictions using a two-way analysis of variance in which the oocyte was included as a random effect (SPSS Statistics 20; IBM, Armonk, NY). Post-hoc testing was with Dunnett’s method, comparing the measured data to each of the predictions.

#### Modeling and Ligand Docking.

Using ClustalW (EMBL-EBI, Cambridge, UK), the protein sequence of the human 5-HT3A subunit (accession: P46098) was aligned with the sequence from the mouse 5-HT_3_A crystal structure (Protein Data Bank ID: 4PIR). Sequence identity between mouse and human 5-HT_3_A was 89.1% (EMBOSS Needle; EMBL-EBI; McWilliam et al., 2013) and alignments were unambiguous. Five pentameric homology models were generated using Modeler 9.13 (Andrej Sali, San Francisco, CA) with default parameters, and the best model was selected using Ramachandran plot analysis. Citral, eucalyptol, and linalool were constructed ab initio in Chem3D Ultra 7.0 (CambridgeSoft, Cambridge, UK) and energy minimized using the MM2 force field. Potential binding sites were identified using a 20-Å docking sphere centered on the *α*-carbon of L320, a residue located within the center of M2; therefore, within each subunit, the docking sphere encompassed the full length of all transmembrane *α*-helices. Docked poses were generated using the GOLD docking program (version 3.0; Cambridge Crystallographic Data Centre, Cambridge, UK) with the GOLDScore function and default settings. For each compound, 10 docking poses were generated, and the poses and predicted hydrogen bonds were visualized with PyMol v1.3 (Schrödinger, New York, NY).

#### Ileum Preparation.

Guinea pig ileum was obtained from adult male guinea pigs (200–300 g). The ileum was cut into 1-cm lengths and mounted longitudinally to a force transducer with a resting tension of 0.5 g in a 10-ml water-jacketed organ bath containing Krebs’ buffer (118 mM NaCl, 4.7 mM KCl, 1.2 mM MgSO_4_, 1.25 mM CaCl_2_, 11 mM glucose, 10 mM HEPES, pH 7.2) continuously aerated with 95% O_2_/5% CO_2_ and kept at 37°C. The ileum segments were allowed to equilibrate for 10 minutes before the experiments were started, and contractile responses were recorded using LabChart 6 (ADInstruments Ltd., Oxford, UK). 5-HT was applied to the serosal layer of the ileum, and contractions were recorded for 10 seconds. Following drug applications, the organ bath was flushed twice with fresh Krebs’ solution, and the ileum was allowed to recover for 10 minutes prior to the next application. Antagonist was preapplied for 10 minutes before coapplying 5-HT. Application of antagonists was not undertaken until two similar (±5%) 5-HT contractions were recorded in the absence of antagonist. Experiments involving animals were approved by the University of Cambridge Animal Welfare and Ethical Review Body (reference: PHARM 004/15).

#### Tracheal Preparation.

The trachea was removed from male Wistar albino rats (250–350 g), cut into 2-cm lengths, and mounted on a force transducer with a resting tension of 1.0 g in a 10-ml water-jacketed organ bath containing modified Tyrode’s solution (136 mM NaCl, 5 mM KCl, 0.98 mM MgCl_2_, 0.36 mM NaH_2_PO_4_, 2 mM CaCl_2_, 11.9 mM NaHCO_3_, 5.5 mM glucose, pH 7.4) continuously aerated with 95% O_2_/5% CO_2_ and kept at 37°C. Contractile responses were recorded using a force transducer (model FT03; Grass Technologies, Quincy, MA) connected to a PC-based data acquisition system (Dataq, PM-1000; DATAQ Instruments Inc., Akron, OH). The experiments were conducted after an equilibration period of 60 minutes and three successive similar contractile responses. Experiments were approved by the local animal ethics committee (protocol number 00084/2014.2).

## Results

### 

#### Effects of Citral, Eucalyptol, and Linalool on 5-HT_3_ Receptor Currents.

**Fig. 2.**
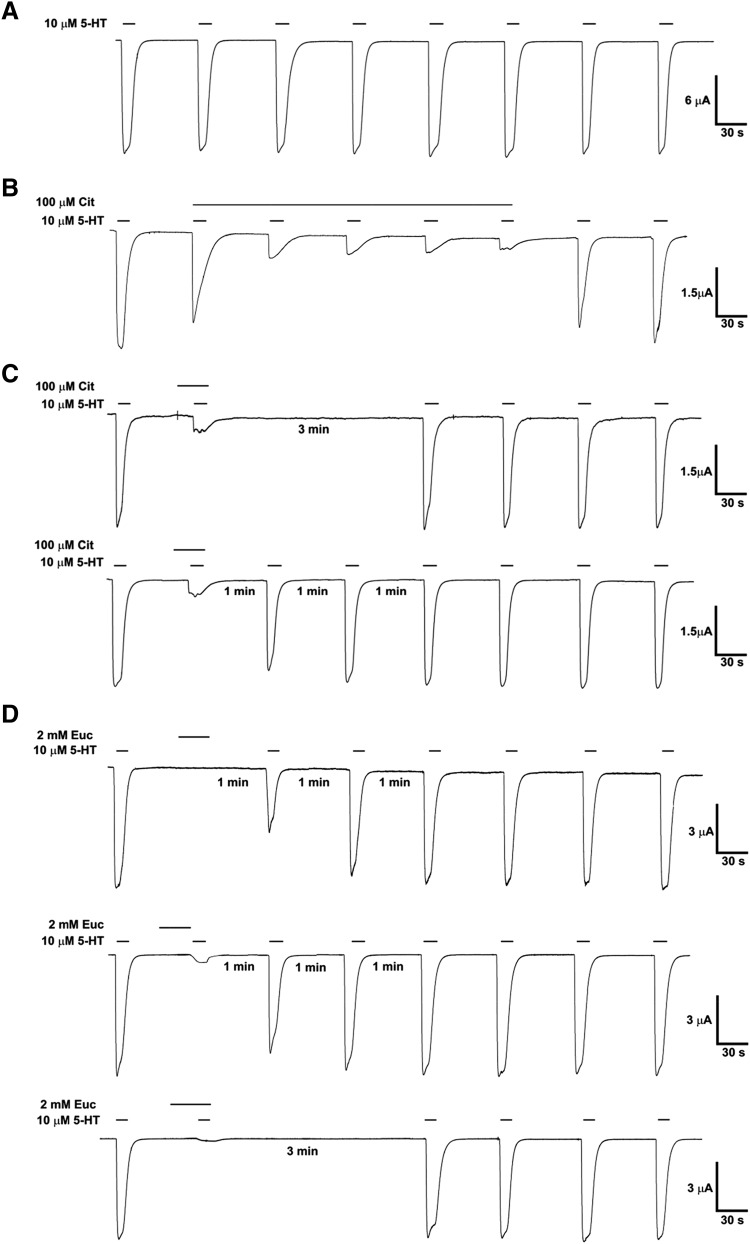

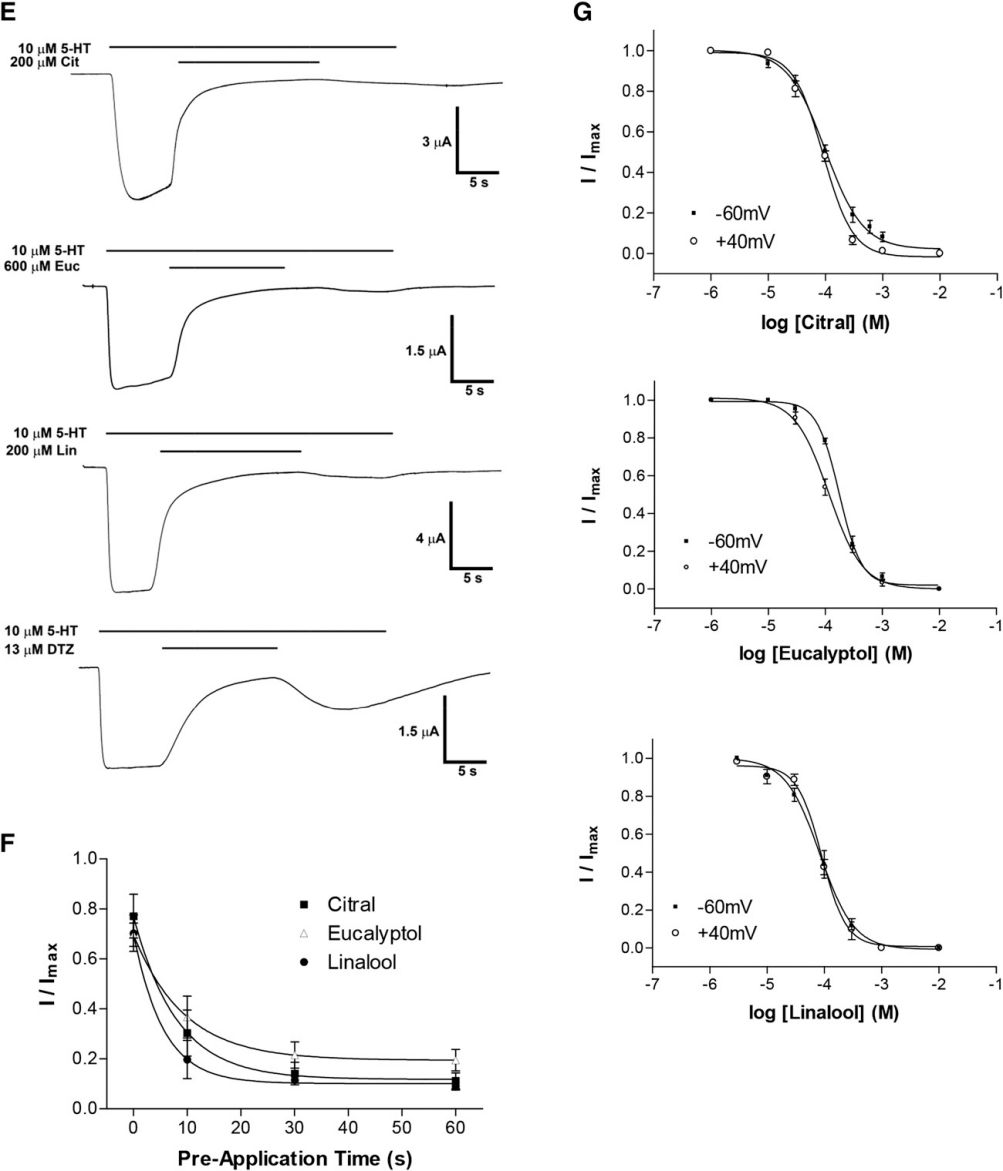
Electrophysiological characterization of the actions of citral (Cit), eucalyptol (Euc), and linalool (Lin) at 5-HT_3_ receptors. (A) Stable currents could be evoked by 10 *µ*M 5-HT applied at 1-minute intervals. (B) At 10 *µ*M 5-HT, stable levels of inhibition by citral, eucalyptol, or linalool were only seen following preapplication but remained stable thereafter [also see (F)]. (C) Following inhibition, responses were slow to recover. For all three compounds, full recovery was achieved after 3 minutes and was independent of the frequency of 5-HT activation during the recovery period. (D) Receptors were inhibited in the closed state as shown by the comparable levels of inhibition seen when the compounds were applied in the absence of 5-HT or during 5-HT application. Inhibition persisted after washout, and the recovery time was unaltered by the timing or frequency of subsequent 5-HT applications. (E) The slow washout of the compounds is highlighted by the absence of a rebound current following the removal of citral, eucalyptol, or linalool. By contrast, rebound was clearly seen following the removal of diltiazem, a channel blocker with faster recovery from inhibition. (F) At a concentration of 300 *µ*M, the oils required 30-second preapplication to stably inhibit the 10 *µ*M 5-HT response. At lower concentrations, the effect of preapplication was less apparent, since the 5-HT response took longer to reach a stable peak current. (G) The level of inhibition at +40 mV or −60 mV was the same for all three compounds.

Neither 5-HT nor the terpenoid oils (Fig. 1) had observable effects on uninjected *Xenopus* oocytes. By contrast, application of 5-HT to *Xenopus* oocytes expressing 5-HT_3_ receptors produced concentration-dependent, rapidly activating, inward currents that slowly desensitized (Fig. 2A). These responses were abolished by the 5-HT_3_ receptor–selective antagonist granisetron (100 nM).

**Fig. 3.**
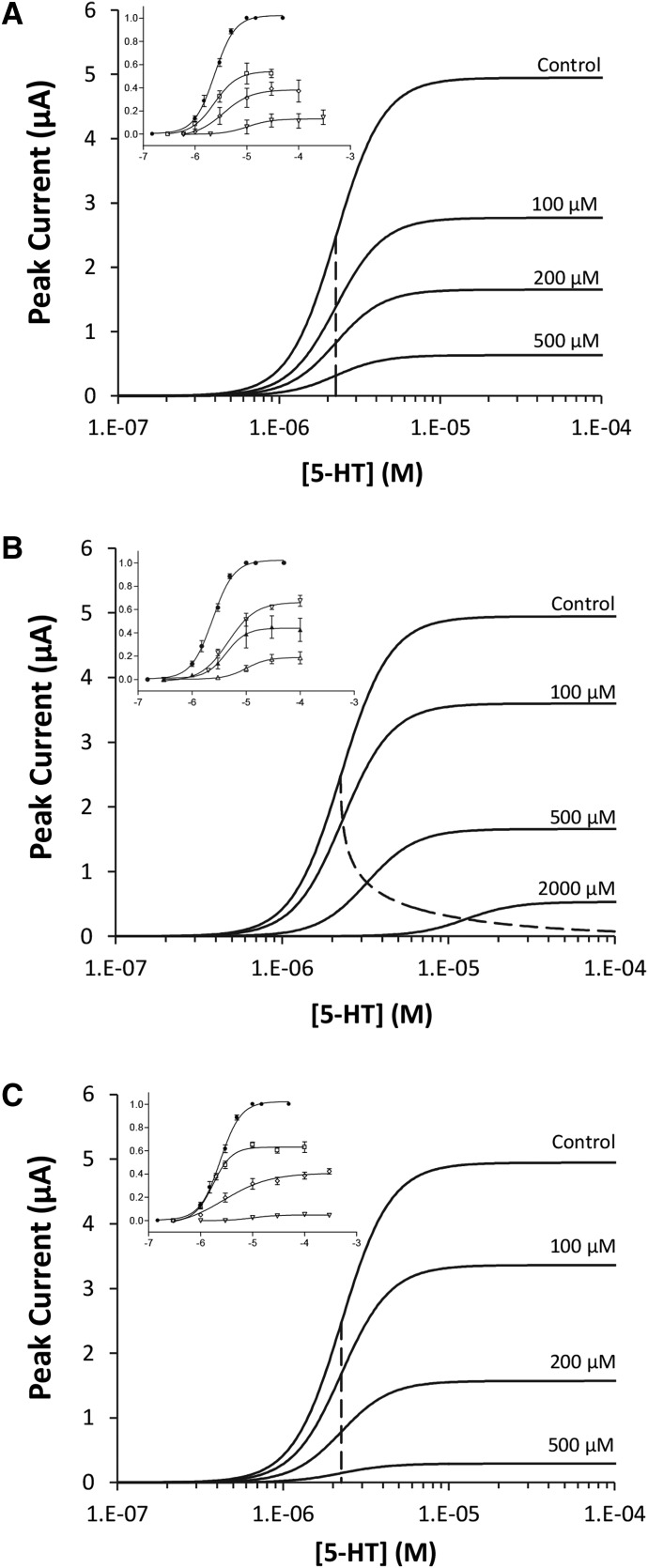
Inhibition of the 5-HT_3_ receptor by citral, eucalyptol, or linalool. 5-HT–induced currents were measured at −60 mV in the absence or presence of various concentrations of citral (A), eucalyptol (B), or linalool (C) following a 30-second preapplication. The data were analyzed as described in the text, and the output of this nonlinear mixed-effects modeling is shown in Table 1. The main features of the structural model are illustrated here. The dotted lines show the effect of the compounds on the EC_50_. The insets show normalized data at the same inhibitor concentrations; for citral and linalool, the apparent shifts in the EC_50_ seen in this normalized data are artifacts caused by the normalization. Additional data at 10, 30, 300, 600, and 1000 *µ*M citral and 200 and 660 *µ*M eucalyptol were also collected, but the curves are omitted for clarity.

Citral, eucalyptol, and linalool inhibited 5-HT–evoked currents in oocytes expressing 5-HT_3_ receptors in a concentration-dependent manner (Figs. 2 and 3) that was unaffected by the clamp potential (Fig. 2G), but had no effect when applied without 5-HT. Preapplication of the oils increased the level of inhibition of the 5-HT response. Figure 2B shows how simultaneous application of 5-HT and citral caused a small reduction in the 5-HT peak current with subsequent responses showing greater inhibition until a stable level of inhibition was achieved. This is also seen in Fig. 2F, where a plot of preapplication time against inhibition shows there was no further increase in the level of inhibition after 30-second preapplication for all three compounds.

Following removal of the compounds, full recovery of 5-HT responses took approximately 3 minutes. This was independent of the frequency of 5-HT application during the recovery period, as shown for citral in Fig. 2C. The rate of recovery from inhibition was similar whether the compounds were applied in the absence of (Fig. 2D, top), immediately prior to (Fig. 2D, middle), or during (Fig. 2D, bottom) 5-HT application. The slow recovery from inhibition was also seen when the three compounds were added and removed in the continuous presence of 5-HT, as no rebound currents were seen, unlike the more rapidly recovering channel blocker diltiazem (Fig. 2E).

These results show that citral, eucalyptol, and linalool inhibit 5-HT_3_ receptors and defined the conditions for subsequent experiments.

#### Quantification and Mechanism of Block.

Studies at varying 5-HT (0.3–300 *µ*M), citral (10, 30, 100, 200, 300, 500, 600, and 1000 *µ*M), eucalyptol (100, 200, 500, 660, and 2000 *µ*M), and linalool (100, 200, 500 *µ*M) concentrations were carried out at −60 mV with a 30-second preapplication of oils and a 3-minute washout.

Peak current responses were reproducible within oocytes but varied substantially between oocytes. A total of 532 peak current values from 55 oocytes were analyzed using nonlinear mixed-effects modeling to characterize and quantify the effects of the compounds. The model included parameters that defined concentration-dependent inhibitor effects on the maximum response (Max_0_) and pEC_50_ of 5-HT.

**Fig. 4.**
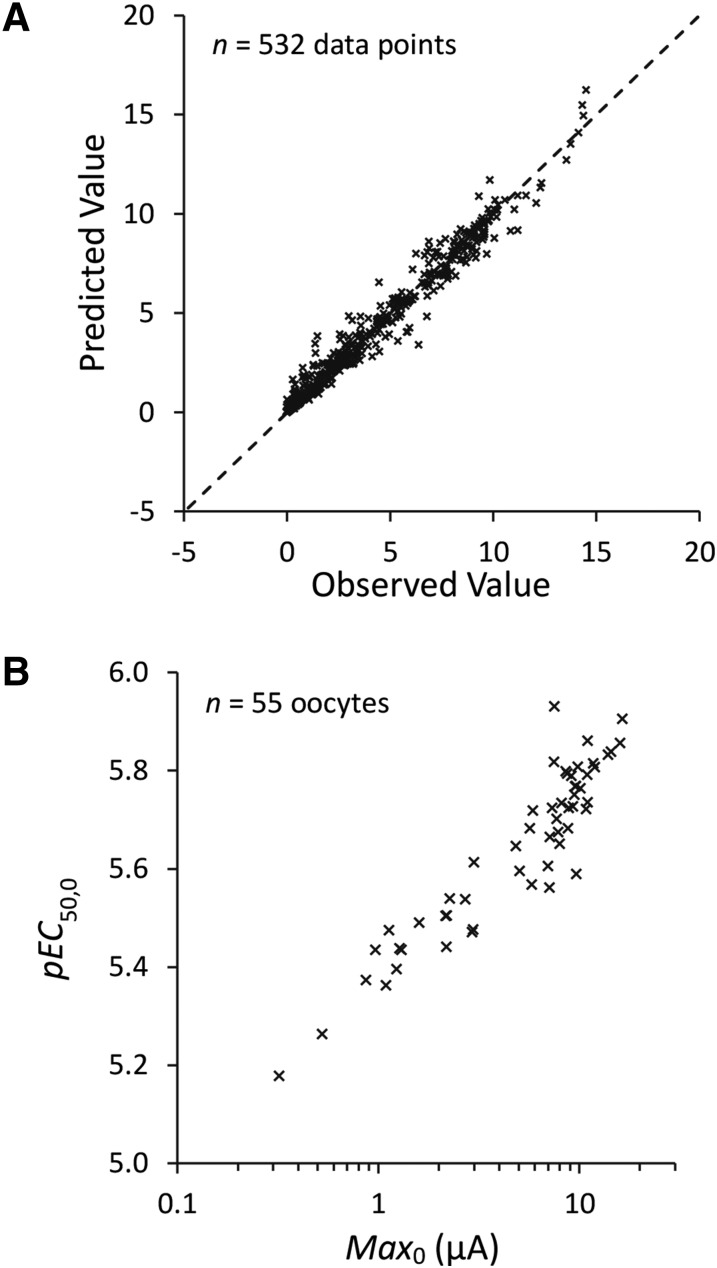
Nonlinear mixed-effects modeling. (A) The relationship between observed and predicted peak current values for the best fit model described in Table 1 (*n* = 532). The values cluster around the line of unity with no apparent systematic deviation, showing that data are well fitted by the model. (B) Modeled Max_0_ and pEC_50,0_ parameter values for individual oocytes (*n* = 55 oocytes). The clear and strong relationship between these parameter estimates (correlation = 0.869 ± 0.054) suggests that oocytes with a high maximum peak current are more sensitive to 5-HT. This relationship is obscured by data normalization.

The results from the best fit model are graphically represented in Fig. 3, with the parameter values shown in Table 1, along with the outcome of hypothesis tests of these parameters. Figure 4A shows a linear relationship between observed and predicted peak current values. The most parsimonious and stable variance-covariance model consisted of seven random effects (Table 1). The analysis with NONMEM also revealed a strong covariance between the maximum response (Max_0_) and pEC_50_ of 5-HT (Fig. 4B: correlation = 0.869 ± 0.054), indicating that oocytes with a higher peak current were more sensitive to 5-HT. Failure to account for this covariance, such as by normalizing data, misrepresents the variance in the responses and could lead to inaccurate conclusions about the effects of the inhibitors on 5-HT pEC_50_ values. For example, the insets in Fig. 3 show normalized representations of the same data revealing apparent shifts in the EC_50_ values for citral and linalool that were not evident when the covariance was taken into account. Furthermore, IC_50_ values derived from these normalized data were lower (e.g., higher potency; citral = 98 *µ*M; eucalyptol = 174 *µ*M; linalool = 83 *µ*M).

In the population of oocytes used (*n* = 55), the 5-HT pEC_50_ was 5.65 ± 0.03 (EC_50_ = 2.25 *µ*M) with a Hill coefficient of 2.94 ± 0.13 (Table 1). This is similar to previous findings and is consistent with strong positive cooperativity between three agonist binding sites (Thompson et al., 2011a; Thompson and Lummis, 2013). Neither citral nor linalool altered the pEC_50_ of 5-HT, but both caused a concentration-dependent reduction in the maximal peak current response (Fig. 3; Table 1). For citral, the pIC_50_ was 3.92 ± 0.05 (IC_50_ = 120 *µ*M), and for linalool, 3.85 ± 0.02 (IC_50_ = 141 *µ*M). Eucalyptol also caused a reduction in the maximal peak current with a pIC_50_ of 3.59 ± 0.11 (IC_50_ = 258 *µ*M), although this was accompanied by a reduction in the pEC_50_ of 5-HT (Fig. 3; Table 1) with a *pA*_2_ and apparent Schild coefficient (defining the shift in the agonist pEC_50_) of 3.09 ± 0.09 and 1.70 ± 0.24, respectively.

These results are consistent with a simple noncompetitive mechanism of action for each compound, but suggest that eucalyptol may have additional effects.

#### Effects on a 5-HT_3_A_T6'S_ Receptor Mutant.

Thymol and carvacrol activate human 5-HT_3_ receptors from a transmembrane binding site (Lansdell et al., 2015). The compounds studied here are structurally similar and could have weak partial agonist activities that are difficult to detect at wild-type receptors. The agonist sensitivity of the 5-HT_3_ receptor can be enhanced by a T6'S substitution in the pore-lining M2 *α*-helix of the 5-HT_3_A subunit (Thompson and Lummis, 2013). We therefore investigated the effects of 5-HT and the terpenoid oils in oocytes expressing the 5-HT_3_A_T6'S_ mutant. A total of 112 peak current responses from 11 different oocytes were appended to the wild-type data set. These experiments comprised control 5-HT concentration-response data and concentration-inhibition data for 2 *µ*M 5-HT. Parameters were included in the model that allowed differences in agonist and inhibitory effects to be quantified and evaluated.

There was no difference in the maximum 5-HT peak current in the wild-type and 5-HT_3_A_T6'S_ receptors [Max_0(T6'S)_/Max_0(WT)_ = 0.98 ± 0.30; *P* = 0.93], but the mutant was more sensitive to agonist as previously reported (Thompson and Lummis, 2013). The 5-HT pEC_50_ was increased by 0.35 ± 0.07. Unlike the effects at wild-type receptors, eucalyptol did not alter the 5-HT pEC_50_ [H_0_: *n_G_*_(EUC/T6'S)_ = 0; *P* = 0.71). When applied alone, citral, eucalyptol, and linalool did not evoke currents in the 5-HT_3_A_T6'S_ receptor mutant, but all abolished 5-HT–induced responses with inhibitory potencies that were reduced by approximately 2-fold when compared with their effects at wild-type receptors (Table 2).

**TABLE 2:** Effect of a T6'S mutation on inhibitor pIC_50_ values

Agonist	ΔpEC_50_	*P* (H_0_: ΔpEC_50_=0)	pEC_50_*^a^*
5-HT	0.35 ± 0.07	3 × 10^−6^	6.00 ± 0.08
Inhibitor*^b^*			
Citral	−0.36 ± 0.10	0.002	3.55 ± 0.11
Eucalyptol	−0.45 ± 0.22	0.046	3.14 ± 0.05
Linalool	−0.34 ± 0.04	5 × 10^−7^	3.51 ± 0.24

^a^ Standard errors were calculated from the errors of the pEC_50_/pIC_50_ and ΔpEC_50_/ΔpIC_50_ values using propagation of error calculations.

^b^ There was no significant difference between the ΔpIC_50_ values for the three inhibitors (*P* = 0.87).

#### Competitive Binding with Granisetron.

**Fig. 5.**
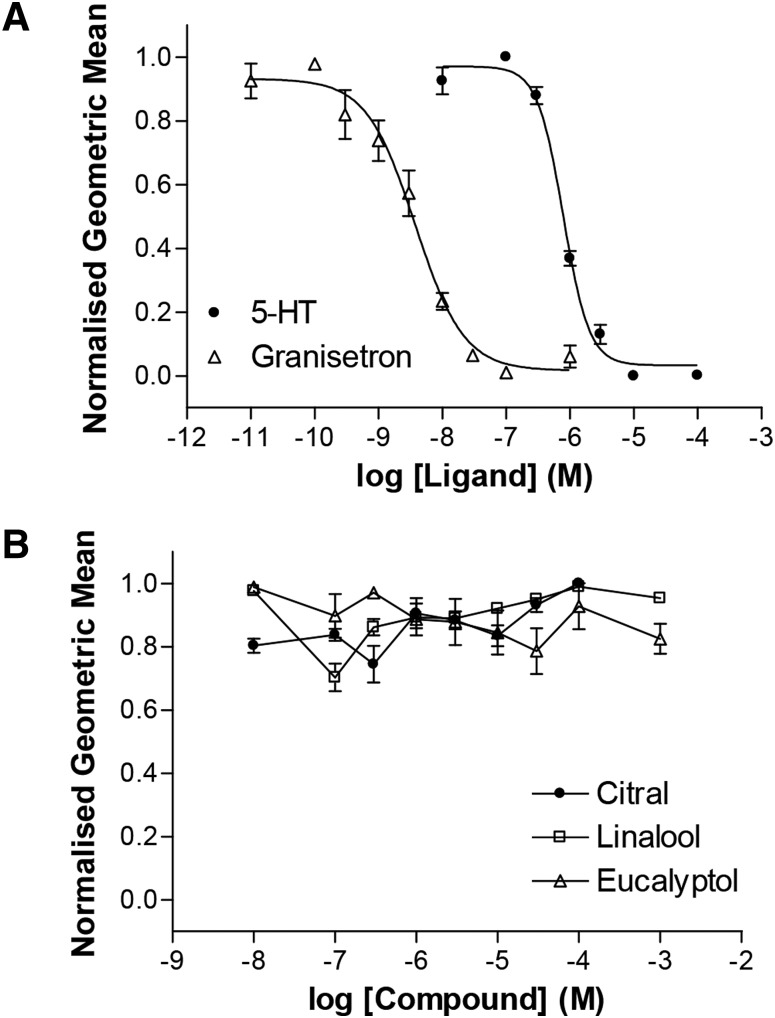
Competition of a fluorescent 5-HT_3_ receptor competitive antagonist. (A) Competition of 10 nM G-FL with the agonist 5-HT (pIC_50_ = 6.10 ± 0.03, *n_H_* = 2.1, IC_50_ = 0.79 *µ*M, *n* = 4) and the competitive antagonist granisetron (pIC_50_ = 8.42 ± 0.03, *n_H_* = 1.1, IC_50_ = 3.8 nM, *n* = 6). (B) The test compounds citral (*n* = 3), eucalyptol (*n* = 3), and linalool (*n* = 3) show no competition with G-FL.

Our analysis provides no evidence that citral or linalool competes with 5-HT at the orthosteric site, whereas the effect of eucalyptol on 5-HT pEC_50_ suggests that this ligand might. To further test for orthosteric interactions, we measured the binding of a fluorescent granisetron derivative (G-FL) together with the three compounds using flow cytometry (Jack et al., 2015). Both 5-HT and granisetron reduced G-FL binding in a concentration-dependent manner (Fig. 5A). Binding of G-FL was unaffected by citral, eucalyptol, or linalool at concentrations up to 1 mM (Fig. 5B).

#### Dual Application of Citral, Eucalyptol, or Linalool in the Presence of Bilobalide or Diltiazem.

We have previously shown that simultaneous application of two drugs can be used to probe the sites of action of channel blockers (Jarvis and Thompson, 2013). Here, we applied each of the oils in the presence of bilobalide or diltiazem, channel-blocking antagonists that bind at the 2'-6' and 7' regions of the 5-HT_3_ receptor pore, respectively (Thompson et al., 2011a).

**Fig. 6.**
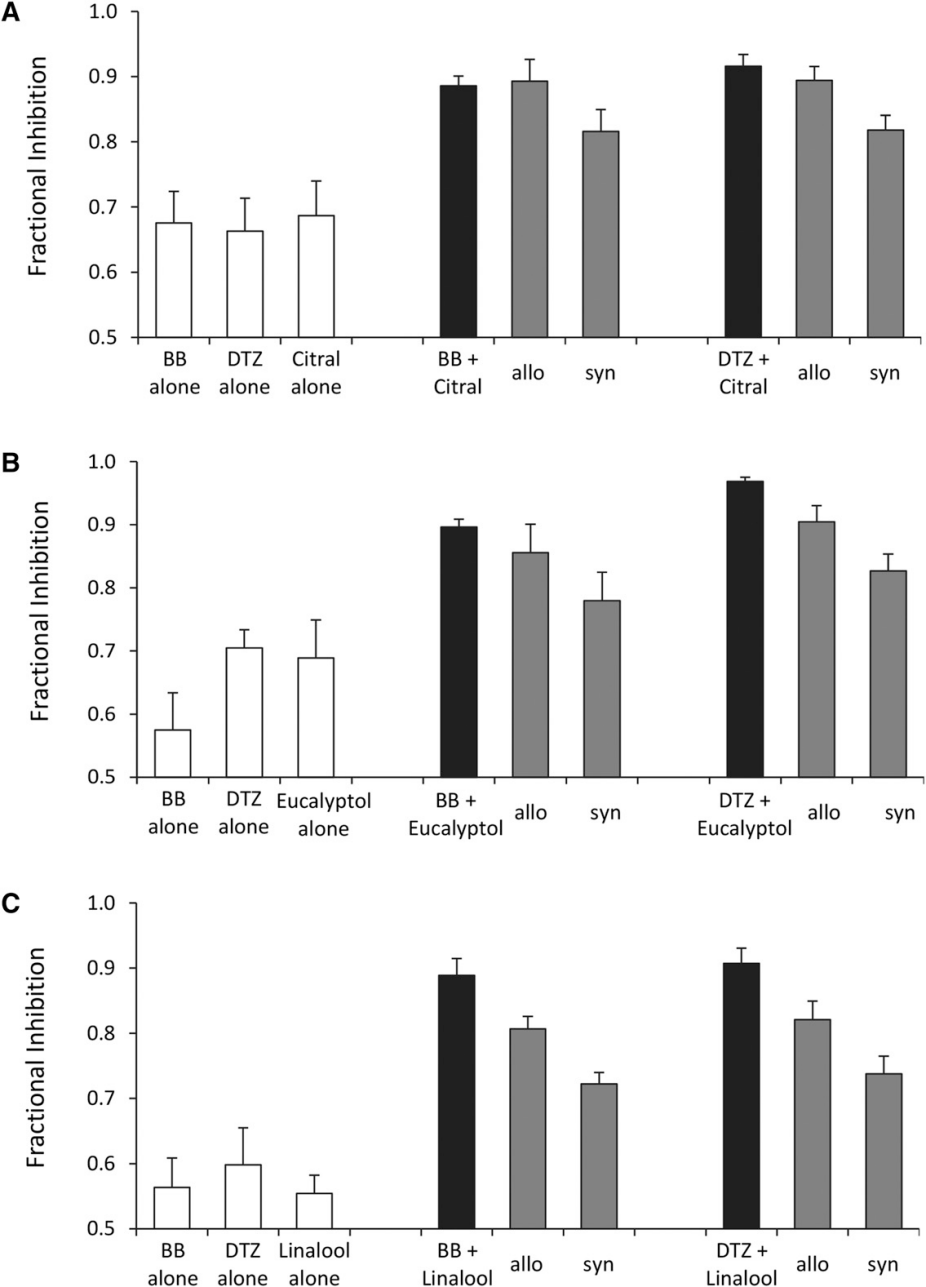
Inhibition of the 5-HT_3_ receptor using a dual-application approach. 5-HT_3_ receptors were activated with a supramaximal (100 *µ*M) concentration of 5-HT. Concentrations of BB, DTZ, and the terpenoids were preselected to inhibit the response by approximately 62% when used alone. Results are shown in the white bars. These same concentrations were then applied in dual combinations, giving results shown in the black bars. The gray bars are the allotopic (allo) and syntopic (syn) predictions based on the levels of inhibition caused by the compounds alone. Data are the mean ± S.E.M. Two-way analysis of variance with Dunnett’s post-hoc test was used (IBM SPSS Statistics 20) to compare actual and predicted dual application responses. (A) Citral (*n* = 7). For BB, *P* = 0.019 (analysis of variance), *P* = 0.95 (H_0_: BB+citral = allo), *P* = 0.031 (H_0_: BB+citral = syn); and for DTZ, *P* = 0.0005 (analysis of variance), *P* = 0.42 (H_0_: DTZ+citral = allo), *P* = 0.0004 (H_0_: DTZ+citral = syn). (B) Eucalyptol (*n* = 6). For BB, *P* = 0.015 (analysis of variance), *P* = 0.38 (H_0_: BB+eucalyptol = allo), *P* = 0.009 (H_0_: BB+eucalyptol = syn); and for DTZ, *P* = 0.00008 (analysis of variance), *P* = 0.013 (H_0_: DTZ+eucalyptol = allo), *P* = 0.00004 (H_0_: DTZ+eucalyptol = syn). (C) Linalool (*n* = 5). For BB, *P* = 0.0006 (analysis of variance), *P* = 0.023 (H_0_: BB+linalool = allo), *P* = 0.0004 (H_0_: BB+linalool = syn); and for DTZ, *P* = 0.003 (analysis of variance), *P* = 0.056 (H_0_: DTZ+linalool = allo), *P* = 0.002 (H_0_: DTZ+linalool = syn). In all experiments, stable levels of inhibition were achieved by applying the compounds for 1 minute before 5-HT was added.

For citral (Fig. 6A), measured dual inhibition was no different from the allotopic predictions for bilobalide (*P* = 0.95) or diltiazem (*P* = 0.42), but was greater than the syntopic predictions (BB, *P* = 0.031; DTZ, *P* = 0.0004). For eucalyptol (Fig. 6B), dual inhibition was similar to the allotopic prediction for bilobalide (*P* = 0.38) but greater than the allotopic prediction for diltiazem (*P* = 0.013). For linalool (Fig. 6C), dual inhibition was greater than the allotopic prediction for both bilobalide (*P* = 0.02) and diltiazem (*P* = 0.06).

These results suggest that citral, eucalyptol, and linalool do not share binding sites with the channel blockers bilobalide or diltiazem, but may enhance their effects.

#### Transmembrane Binding Sites.

**Fig. 7.**
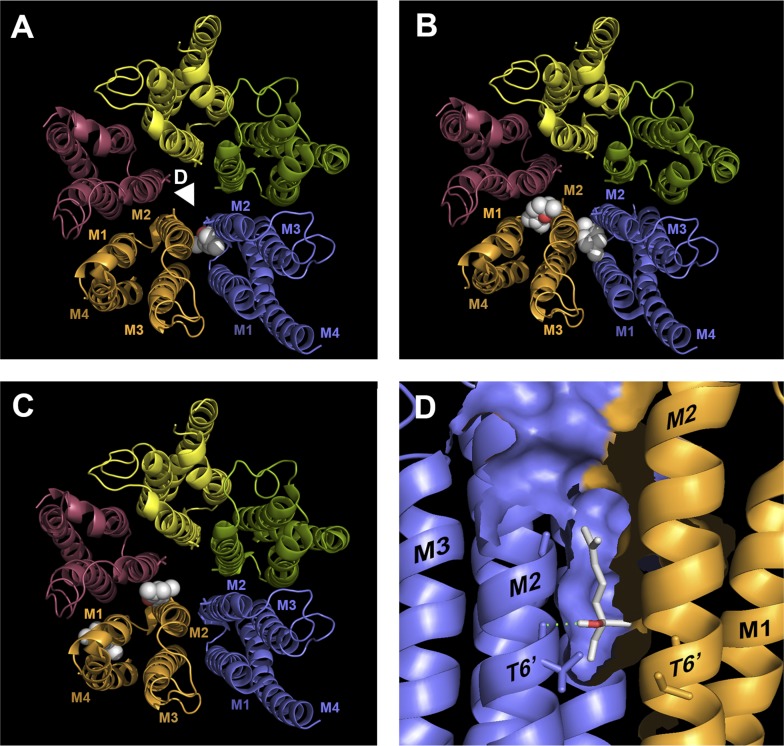
Predicted binding locations for citral, eucalyptol, and linalool in a homology model of the human 5-HT_3_ receptor. Using a loosely defined binding site (see *Materials and Methods*), 10 docked poses were generated for each ligand. Examples from the docked pose clusters (sphere representation) are shown at each of the predicted binding sites. The transmembrane domains of the five subunits that form the functional 5-HT_3_ receptor are shown as different colored ribbons viewed from the extracellular side, with both the intracellular and extracellular domains removed for clarity. (A) A single binding site was predicted for linalool with the docked pose cluster differing by only 2.29 Å root-mean-square deviation. The white arrowhead indicates the origin from which (D) is viewed. (B) Two sites were predicted for eucalyptol, but were similarly located at the boundaries of adjacent subunits. (C) For citral, two potential binding sites were identified, a major (6/10) site at the interface of adjacent subunits and a minor site located at the lipid-exposed interface at the intracellular ends of M1 and M4. (D) In four of the 10 docked poses for linalool, PyMol 1.3 predicted hydrogen bonds (blue dotted line) between the ligand’s terminal hydroxyl and the backbone of the channel-lining 6' Thr residue.

Given the lipophilicity of the oils and their noncompetitive mechanism of action, we sought to identify potential binding sites in the transmembrane domain using in silico ligand docking in a homology model of the human 5-HT_3_ receptor. Using a loosely defined binding site radius of 20 Å that encompassed the whole of the transmembrane domain, the major binding cavity for all of the ligands was predicted to be at the interface of two adjacent subunits, between M1-M2 of the principal subunit and M2-M3 of the complementary subunit. For linalool, all 10 docked poses were similarly orientated at the intersubunit interface within only a 2.29-Å root-mean-square deviation (Fig. 7A). Eucalyptol docked at two locations, both of which were clustered at the same site between two adjacent subunits (Fig. 7B). For citral, two sites were predicted, with the major docked pose cluster (6/10) at the same intersubunit cavity, and a minor site (4/10) at the lipid-exposed intracellular interface of the M1 and M4 *α*-helices (Fig. 7C). Linalool was the only compound predicted to make hydrogen bond contacts with the protein (in 4/10 docked poses), which were between the hydroxyl of linalool and the backbone carbonyl of Thr6' in M2 (Fig. 7D).

These results suggest that all three compounds could bind to a transmembrane cavity located at the interface of adjacent subunits.

#### Physiologic Effects.

*L. alba* is used medicinally throughout Central and South America as a means of alleviating gastrointestinal discomfort and for respiratory ailments (Hennebelle et al., 2008). Here, we analyzed an OELa by LC-MS and detected that the main compounds were the terpenoids citral (75.9%; 41.8% geranial + 34.1% neral), 1-limoneno (9.8%), carvone (8.9%), gamma-terpinene (2.0%), and benzene [1-methyl-3-(1-methylethyl; 1.0%].

**Fig. 8.**
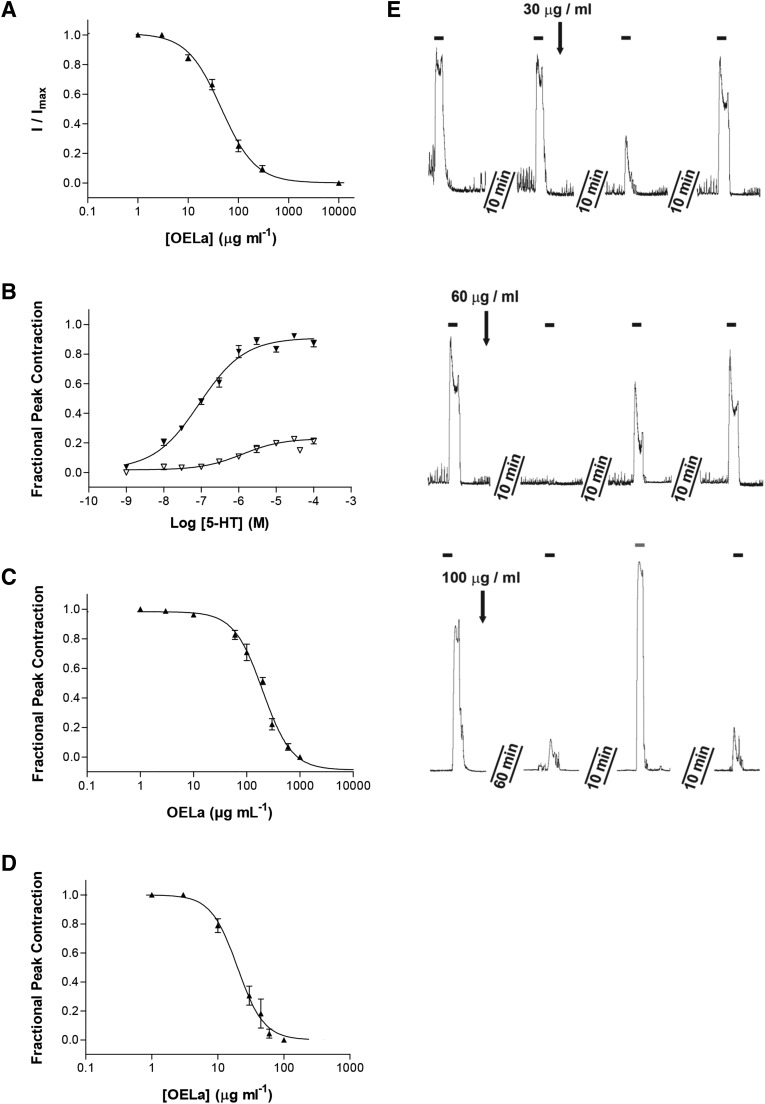
The effects of OELa. (A) Concentration inhibition of 2 *µ*M 5-HT–induced currents by OELa in *Xenopus* oocytes expressing the 5-HT_3_ receptor (*n* = 5). (B) Rat tracheal contraction in response to 5-HT in the absence (▾, *n* = 6) and following a 10-minute preapplication with 10 *µ*M granisetron (▽, *n* = 3). (C) Concentration-inhibition of the 10 *µ*M 5-HT–evoked tracheal contractile response by OELa (*n* = 4). (D) Inhibition by OELa of 10 *µ*M 5-HT–evoked guinea pig ileum contractions (*n* = 6). (E). Example recordings from 5-HT–evoked (black bars) contractions of guinea pig ileum and their inhibition by 30, 60, and 100 *µ*g ml^−1^ OELa. At concentrations ≥60 *µ*g ml^−1^ OELa, the 5-HT–evoked contractions became increasingly slow to recover. Following a control 5-HT response (0.6 *µ*M, black bar), the addition of 100 *µ*g ml^−1^ OELa continued to inhibit the 5-HT–evoked contractions 50 minutes later, although an acetylcholine (1 *µ*M, gray bar) response was unaltered. Parameters defining the curves are shown in the text.

To determine whether the properties of this essential oil could be physiologically relevant, we measured its effects on 5-HT_3_ receptors expressed in oocytes and on smooth muscle contractions in rat trachea and guinea pig ileum. At 5-HT_3_ receptors expressed in oocytes, OELa had no effect when applied alone, but fully and reversibly inhibited the 2 *µ*M 5-HT response with an IC_50_ of 45 *µ*g ml^−1^ (*n* = 5; Fig. 8A). In rat trachea, 5-HT-induced contractions were concentration-dependent and substantially inhibited by 10 µM of the 5-HT3 receptor antagonist granisetron (Fig. 8B). OELa also abolished contractions induced by 10 *µ*M 5-HT with an IC_50_ of 200 *µ*g ml^−1^ (Fig. 8C). 5-HT–evoked contractions of guinea pig ileum were also inhibited by granisetron, and OELa had an IC_50_ of 20 *µ*g ml^−1^ (*n* = 6; Fig. 8D). In both rat trachea and guinea pig ileum, recovery of contractions required extended washes, particularly at higher concentrations (Fig. 8E).

These results show that that OELa has a high citral content and inhibits 5-HT_3_ receptors expressed in *Xenopus* oocytes and 5-HT–induced smooth muscle contraction in the trachea and ileum.

## Discussion

This study describes the inhibitory effects of citral, eucalyptol, and linalool on human 5-HT_3_ receptors. These terpenoids can be added to a growing list of structurally related compounds that modulate a wide range of voltage- and ligand-gated ion channels, including 5-HT_3_ receptors and their related vertebrate, invertebrate, and prokaryotic homologs (Hales and Lambert, 1991; Barann et al., 2000; Hall et al., 2004; Garcia et al., 2006; Ashoor et al., 2013; Walstab et al., 2014; Lansdell et al., 2015; Ziemba et al., 2015).

All of the essential oils inhibited 5-HT–mediated currents with IC_50_ values in the micromolar range. Non-competitive antagonism was shown by them causing a concentration-dependent reduction in the maximal 5-HT response and by not competing with the fluorescent antagonist G-FL. The Hill coefficients for the reduction in the 5-HT response suggested that there may be multiple binding sites with strong cooperativity for linalool (*n_H_* = 2.2), weak cooperativity for citral (*n_H_* = 1.3), and no cooperativity for eucalyptol (*n_H_* = 1.0). There was evidence that, at higher concentrations, eucalyptol also caused a rightward shift in the EC_50_ of 5-HT (*pA*_2_ = 3.09), indicating an additional inhibitory mechanism. A competitive mechanism is conceivable, although not compelling, given that the shift had a Schild coefficient of 1.7 and that eucalyptol failed to compete with G-FL. However, given the different binding orientations of 5-HT and granisetron in cocrystal structures, it is possible that an inhibitor could compete with one of these ligands but not the other (Colquhoun, 2007; Kesters et al*.,* 2013). At the T6'S mutants, there was no evidence of a eucalyptol-induced change in agonist EC_50_. This is probably because this effect occurs at higher concentrations (*pA*_2_ = 3.09) than the reduction in maximal current (pIC_50_ = 3.59), and the overall potency of the terpenoids was reduced at these mutants.

Mixed-effects modeling also revealed a strong correlation between the maximal peak current and pEC_50_ (Fig. 5B). Since receptor expression is difficult to control, many investigators normalize data to facilitate comparisons between experiments. However, if the implicit assumption that agonist response is independent of expression levels is not justified, then data normalization may lead to inaccurate and misleading conclusions. In the current study, a particular advantage of the nonlinear mixed-effects modeling was that variant maximal current responses could be modeled as a random effect, thereby generating more accurate quantitative conclusions (normalization yielded higher IC_50_ values, suggesting that this method of analysis overestimated the potency of the compounds) and revealing unexpected biologic phenomena (such as the covariance of the EC_50_ and Max_0_). This modeling approach was also able to identify pleiotropic drug effects (e.g., eucalyptol), for which the properties of the compounds we used were particularly well suited. Therefore, the data we present clearly highlight the superior value of nonlinear mixed-effects modeling, revealing phenomena that would otherwise have been missed using the standard procedure of normalizing data. Nonlinear mixed-effects modeling is often used for pharmacokinetic-pharmacodynamic data analysis, but has broad applicability in other quantitative pharmacological studies (Mould and Upton, 2013). For example, by modeling all data simultaneously (e.g., by incorporating both agonist concentration-activation and antagonist concentration-inhibition data), it generates a more comprehensive model of drug action and a statistically more powerful framework within which to evaluate specific hypotheses. This is particularly valuable when it is important to minimize use of resources, such as experimental animals or scarce test compounds.

Previously, we used a dual-application method to determine whether channel blockers have overlapping or independent binding sites within the 5-HT_3_ receptor pore (Thompson et al., 2011b; Jarvis and Thompson, 2013). Results suggesting that bilobalide, ginkgolide B, and picrotoxinin shared binding sites and that diltiazem bound elsewhere were later confirmed by mutating channel-lining residues (Thompson et al., 2011a). Dual application of citral with bilobalide or diltiazem caused inhibition consistent with the allotopic model, indicating that citral binds at distinct sites to these two channel blockers. Eucalyptol and linalool caused dual inhibition at levels significantly greater than the syntopic predictions, indicating that these compounds also bind to distinct sites. However, dual inhibition exceeded the allotopic prediction, suggesting that eucalyptol and linalool may allosterically modulate binding of bilobalide and diltiazem. Alternatively, as the allotopic model assumes that the inhibitors do not modify the action of the agonist, it is possible that a reduced sensitivity to 5-HT could contribute to this effect, a possibility that is supported by the rightward shift in the 5-HT EC_50_ caused by eucalyptol.

The lipophilic nature of the oils suggests that the binding sites of citral, eucalyptol, and linalool are located in the transmembrane region, consistent with the slow wash-in and washout that is observed when compounds must first diffuse into membranes before reaching their target (Turina et al., 2006). This hypothesis is supported by our electrophysiology and flow cytometry data, which indicate a noncompetitive mechanism of action, and by the nonoverlapping binding sites predicted by our dual-application experiments. To probe for potential binding sites, we used homology modeling and docking, which predicted that all three oils share a common binding site located in a cavity between the transmembrane *α*-helices of adjacent subunits. The terpenoids carvacrol and thymol have been proposed to bind in a similar cavity within the 5-HT_3_ receptor, and this location is also conserved among other Cys-loop receptors (Lansdell et al., 2015). Crystal structures of the invertebrate Cys-loop receptor glutamate-gated chloride channel reveal an equivalent intersubunit cavity occupied by ivermectin, and residues at this location are implicated in ivermectin binding at GABA_A_ and glycine receptors (Lynagh and Lynch, 2012). A similar binding site has been identified for propofol at a prokaryotic homolog (*Gloeobacter* violaceus ligand-gated ion channel) and other eukaryotic Cys-loop receptors (Nury et al., 2011; Jayakar et al., 2013; Yip et al., 2013; Lynagh and Laube, 2014). The similar activities of these compounds across the Cys-loop receptor family suggest that this region is widely conserved and could be an amenable target for novel allosteric modulators (Corradi et al., 2011; Howard et al., 2011; Trattnig et al., 2012). To date, 5-HT_3_ receptor antagonists have been typically used to prevent nausea and vomiting, but the association of these receptors with other disorders, such as irritable bowel syndrome, anxiety, and diabetic neuropathy, suggests that there may be scope for other therapeutic applications (Walstab et al., 2010; Silva et al., 2015).

The structurally related compounds carvacrol and thymol are also partial agonists at the 5-HT_3_ receptor (Lansdell et al., 2015; Ziemba et al., 2015). As low-efficacy partial agonism can sometimes be overlooked and mistaken as antagonism [e.g., the 5-HT_3_ ligand quipazine (Thompson and Lummis, 2013)], we tested citral, eucalyptol, and linalool for agonist activity using a 5-HT_3_ receptor mutant with enhanced agonist sensitivity. Substitution of the 5-HT_3_A subunit channel-lining 6' Thr with the equivalent 6' Ser from the 5-HT_3_B subunit creates a hypersensitive mutant with increased sensitivity to a range of 5-HT_3_ agonists (Thompson and Lummis, 2013). Consistent with previous reports, our results reveal a 2-fold increase in 5-HT sensitivity in the T6'S mutant. However—with citral, eucalyptol, and linalool—no agonist action, or potentiation of the 5-HT response, was observed at the mutant receptor, confirming that they are antagonists. In silico docking predicted that the 6' Thr residue may establish a hydrogen-bond interaction with linalool, although the interaction was with the backbone carbonyl and therefore unlikely to be greatly affected by amino acid substitution. Indeed, a 2-fold reduction in the potency of all the inhibitors at 5-HT_3_A_T6'S_ mutants suggests a nonspecific effect rather than a modification of hydrogen-bond interactions for linalool alone.

Several plant extracts are commonly used to treat gastrointestinal discomfort and respiratory disorders, and their components, such as citral and menthol, are reported to have relaxant effects on gut smooth muscle (Tangpu and Yadav, 2006; Hennebelle et al., 2008, Devi et al., 2011; Walstab et al., 2014). Here, we show that OELa, an extract from *L. alba*, inhibits 5-HT_3_ receptors expressed in oocytes (IC_50_ = 45 *µ*g ml^−1^) and 5-HT–evoked contractions in both rat trachea (IC_50_ = 200 *µ*g ml^−1^) and guinea pig ileum (IC_50_ = 20 *µ*g ml^−1^). Using LC-MS, we found the principal component of this oil was citral (75.9%), which could account for these effects; the IC_50_ of OELa at expressed receptors is 45 *µ*g ml^−1^, which contains ∼220 *µ*M citral and is close to the IC_50_ of citral alone (120 *µ*M). It has been reported that 5-HT–mediated smooth muscle contractions in the trachea and ileum are partially regulated by 5-HT_2_, 5-HT_3_, and 5-HT_4_ receptors. Our results with the antagonist granisetron confirm a role for 5-HT_3_ receptors, similar to previous reports that used 5-HT_3_ receptor antagonists to inhibit 5-HT–mediated smooth muscle contractions (Rocha e Silva et al., 1953; Tuladhar et al., 1997; Fernandez-Rodriguez et al., 2010; Kelley et al., 2014). Our results may therefore provide a mechanism for the medicinal use of these oils for relieving gastrointestinal and respiratory ailments. The IC_50_ values we report here are comparable to those for similar terpenoid compounds, such as menthol (163 *µ*M), and it has been suggested that oral administration of this compound could reach an equivalent concentration in vivo (Ashoor et al., 2013). However, to achieve this concentration in rat brain, the required intraperitoneal dose of menthol was 100 mg/kg, considerably higher than doses used for human medicinal products (Pan et al., 2012). Therefore, in humans, it is unlikely that systemic administration of typical doses would result in concentrations that are active at 5-HT_3_ receptors, although it is possible that higher local concentrations following topical administration (e.g., in airway, skin, or gut) could reach pharmacologically active levels (Falk-Filipsson et al., 1993). For most terpenoids, including linalool and eucalyptol, blood and tissue concentrations have not been reported, but toxicological studies suggest that many are well tolerated (Oz et al., 2015). This suggests that there is still scope for therapeutic applications, with synthetic modification possibly providing a means for improving their potency and receptor selectivity.

In summary, we used nonlinear mixed-effects modeling to show that the oils citral, eucalyptol, and linalool inhibit homomeric 5-HT_3_ receptors via noncompetitive mechanisms. Both electrophysiology and flow cytometry point to binding locations that do not overlap with the orthosteric binding site, whereas our dual-application experiments suggest actions that are mediated from outside the pore. Docking predicts a transmembrane binding site located between the *α*-helices of adjacent subunits, and is supported by the binding of related compounds to similar allosteric sites identified in both 5-HT_3_ and other members of this ligand-gated ion channel family. These results demonstrate the value of analyzing data using nonlinear mixed-effects modeling and further highlight a conserved transmembrane binding site as a potential target for the development of novel allosteric ligands.

## Abbreviations

BB: bilobalide
DMEM: Dulbecco’s modified Eagle’s medium
DTZ: diltiazem
ELIC: *Erwinia* chrysanthemi ligand-gated ion channel
G-FL: granisetron-fluorescein
GLIC: Gloeobacter violaceus ligand-gated ion channel
GluCl: glutamate-gated chloride channel
HEK293: human embryonic kidney 293
LC-MS: gas-liquid chromatography coupled to mass spectrometry
OELa: essential oil extracts from *Lippia alba*
PRED: predicted agonist-induced response
RDL: resistance-to-dieldrin channels
RUV: residual unexplained variance

## References

[B1] Abdel-Aziz H Windeck T Ploch M Verspohl EJ (2006) Mode of action of gingerols and shogaols on 5-HT_3_ receptors: binding studies, cation uptake by the receptor channel and contraction of isolated guinea-pig ileum. Eur J Pharmacol 530:136–143. 16364290 10.1016/j.ejphar.2005.10.049

[B2] Ashoor A Nordman JC Veltri D Yang KH Shuba Y Al Kury L Sadek B Howarth FC Shehu A Kabbani N (2013) Menthol inhibits 5-HT_3_ receptor-mediated currents. J Pharmacol Exp Ther 347:398–409. 23965380 10.1124/jpet.113.203976

[B3] Barann M Dilger JP Bönisch H Göthert M Dybek A Urban BW (2000) Inhibition of 5-HT_3_ receptors by propofol: equilibrium and kinetic measurements. Neuropharmacology 39:1064–1074. 10727717 10.1016/s0028-3908(99)00205-1

[B4] Caputi L Aprea E (2011) Use of terpenoids as natural flavouring compounds in food industry. Recent Pat Food Nutr Agric 3:9–16. 21114471 10.2174/2212798411103010009

[B5] Chiara DC Jayakar SS Zhou X Zhang X Savechenkov PY Bruzik KS Miller KW Cohen JB (2013) Specificity of intersubunit general anesthetic-binding sites in the transmembrane domain of the human α1β3γ2 γ-aminobutyric acid type A (GABAA) receptor. J Biol Chem 288:19343–19357. 23677991 10.1074/jbc.M113.479725 PMC3707639

[B6] Colquhoun D (2007) Why the Schild method is better than Schild realised. Trends Pharmacol Sci 28:608–614. 18023486 10.1016/j.tips.2007.09.011

[B7] Corradi J Andersen N Bouzat C (2011) A novel mechanism of modulation of 5-HT₃A receptors by hydrocortisone. Biophys J 100:42–51. 21190655 10.1016/j.bpj.2010.10.046 PMC3010835

[B8] Devi RC Sim SM Ismail R (2011) Spasmolytic effect of citral and extracts of *Cymbopogon citratus* on isolated rabbit ileum. J Smooth Muscle Res 47:143–156. 22104376 10.1540/jsmr.47.143

[B9] Falk-Filipsson A Löf A Hagberg M Hjelm EW Wang Z (1993) d-limonene exposure to humans by inhalation: uptake, distribution, elimination, and effects on the pulmonary function. J Toxicol Environ Health 38:77–88. 8421324 10.1080/15287399309531702

[B10] Fernandez-Rodriguez S Broadley KJ Ford WR Kidd EJ (2010) Increased muscarinic receptor activity of airway smooth muscle isolated from a mouse model of allergic asthma. Pulm Pharmacol Ther 23:300–307. 20347047 10.1016/j.pupt.2010.03.001

[B11] García DA Bujons J Vale C Suñol C (2006) Allosteric positive interaction of thymol with the GABA_A_ receptor in primary cultures of mouse cortical neurons. Neuropharmacology 50:25–35. 16185724 10.1016/j.neuropharm.2005.07.009

[B12] Hales TG Lambert JJ (1991) The actions of propofol on inhibitory amino acid receptors of bovine adrenomedullary chromaffin cells and rodent central neurones. Br J Pharmacol 104:619–628. 1665745 10.1111/j.1476-5381.1991.tb12479.x PMC1908220

[B13] Hall AC Turcotte CM Betts BA Yeung WY Agyeman AS Burk LA (2004) Modulation of human GABA_A_ and glycine receptor currents by menthol and related monoterpenoids. Eur J Pharmacol 506:9–16. 15588619 10.1016/j.ejphar.2004.10.026

[B50] Hassaine G Deluz C Grasso L Wyss R Tol MB Hovius R Graff A Stahlberg H Tomizaki T Desmyter A Moreau C Li XD Poitevin F Vogel H Nury H (2014) X-ray structure of the mouse serotonin 5-HT_3_ receptor. Nature 512:276–281. 25119048 10.1038/nature13552

[B14] Hawthorne R Cromer BA Ng HL Parker MW Lynch JW (2006) Molecular determinants of ginkgolide binding in the glycine receptor pore. J Neurochem 98:395–407. 16805834 10.1111/j.1471-4159.2006.03875.x

[B16] Heimes K Hauk F Verspohl EJ (2011) Mode of action of peppermint oil and (-)-menthol with respect to 5-HT_3_ receptor subtypes: binding studies, cation uptake by receptor channels and contraction of isolated rat ileum. Phytother Res 25:702–708. 21077259 10.1002/ptr.3316

[B17] Hennebelle T Sahpaz S Joseph H Bailleul F (2008) Ethnopharmacology of *Lippia alba*. J Ethnopharmacol 116:211–222. 18207682 10.1016/j.jep.2007.11.044

[B18] Howard RJ Murail S Ondricek KE Corringer PJ Lindahl E Trudell JR Harris RA (2011) Structural basis for alcohol modulation of a pentameric ligand-gated ion channel. Proc Natl Acad Sci USA 108:12149–12154. 21730162 10.1073/pnas.1104480108 PMC3141919

[B51] Sali A Blundell TL (1993) Comparative protein modelling by satisfaction of spatial restraints. J Mol Biol 234:779–815. 8254673 10.1006/jmbi.1993.1626

[B19] Jack T Simonin J Ruepp MD Thompson AJ Gertsch J Lochner M (2015) Characterizing new fluorescent tools for studying 5-HT₃ receptor pharmacology. Neuropharmacology 90:63–73. 25460187 10.1016/j.neuropharm.2014.11.007

[B20] Jarvis GE Thompson AJ (2013) A golden approach to ion channel inhibition. Trends Pharmacol Sci 34:481–488. 23972927 10.1016/j.tips.2013.07.004 PMC3769878

[B21] Jayakar SS Dailey WP Eckenhoff RG Cohen JB (2013) Identification of propofol binding sites in a nicotinic acetylcholine receptor with a photoreactive propofol analog. J Biol Chem 288:6178–6189. 23300078 10.1074/jbc.M112.435909 PMC3585054

[B22] Kelley SP Walsh J Kelly MC Muhdar S Adel-Aziz M Barrett ID Wildman SS (2014) Inhibition of native 5-HT3 receptor-evoked contractions in guinea pig and mouse ileum by antimalarial drugs. Eur J Pharmacol 738:186–191. 24886883 10.1016/j.ejphar.2014.05.043

[B23] Kessler A Sahin-Nadeem H Lummis SC Weigel I Pischetsrieder M Buettner A Villmann C (2014) GABA(_A_) receptor modulation by terpenoids from Sideritis extracts. Mol Nutr Food Res 58:851–862. 24273211 10.1002/mnfr.201300420 PMC4384808

[B24] Kesters D Thompson AJ Brams M van Elk R Spurny R Geitmann M Villalgordo JM Guskov A Danielson UH Lummis SC (2013) Structural basis of ligand recognition in 5-HT_3_ receptors. EMBO Rep 14:49–56. 23196367 10.1038/embor.2012.189 PMC3537142

[B25] Lansdell SJ Sathyaprakash C Doward A Millar NS (2015) Activation of human 5-hydroxytryptamine type 3 receptors via an allosteric transmembrane site. Mol Pharmacol 87:87–95. 25338672 10.1124/mol.114.094540

[B26] Lynagh T Laube B (2014) Opposing effects of the anesthetic propofol at pentameric ligand-gated ion channels mediated by a common site. J Neurosci 34:2155–2159. 24501356 10.1523/JNEUROSCI.4307-13.2014 PMC6608535

[B27] Lynagh T Lynch JW (2012) Ivermectin binding sites in human and invertebrate Cys-loop receptors. Trends Pharmacol Sci 33:432–441. 22677714 10.1016/j.tips.2012.05.002

[B28] McWilliam H Li W Uludag M Squizzato S Park YM Buso N Cowley AP Lopez R (2013) Analysis Tool Web Services from the EMBL-EBI. Nucleic Acids Res 41:W597–W600. 23671338 10.1093/nar/gkt376 PMC3692137

[B29] Moore NA Sargent BJ Manning DD Guzzo PR (2013) Partial agonism of 5-HT_3_ receptors: a novel approach to the symptomatic treatment of IBS-D. ACS Chem Neurosci 4:43–47. 23342199 10.1021/cn300166c PMC3548414

[B30] Mould DR Upton RN (2013) Basic concepts in population modeling, simulation, and model-based drug development-part 2: introduction to pharmacokinetic modeling methods. CPT Pharmacometrics Syst Pharmacol 2:e38. 23887688 10.1038/psp.2013.14 PMC3636497

[B31] Nury H Van Renterghem C Weng Y Tran A Baaden M Dufresne V Changeux JP Sonner JM Delarue M Corringer PJ (2011) X-ray structures of general anaesthetics bound to a pentameric ligand-gated ion channel. Nature 469:428–431. 21248852 10.1038/nature09647

[B32] Oz M Lozon Y Sultan A Yang KH Galadari S (2015) Effects of monoterpenes on ion channels of excitable cells. Pharmacol Ther 152:83–97. 25956464 10.1016/j.pharmthera.2015.05.006

[B33] Pan R Tian Y Gao R Li H Zhao X Barrett JE Hu H (2012) Central mechanisms of menthol-induced analgesia. J Pharmacol Exp Ther 343:661–672. 22951274 10.1124/jpet.112.196717

[B34] Rocha e Silva M Valle JR Picarelli P (1953) A pharmacological analysis of the mode of action of serotonin (5-hydroxytryptamine) upon the guinea-pig ileum. Br Pharmacol Chemother 8:378–388. 10.1111/j.1476-5381.1953.tb01333.x PMC1509359 13115625

[B49] Silva M Martins D Tavares I Morgado C (2015) Inhibition of spinal 5-HT_3_R reverted diabetes-induced mechanical hypersensitivity in a GABA_A_R-mediated neurotransmission-dependent manner. Neuroscience 304:228–239. 26210577 10.1016/j.neuroscience.2015.07.050

[B35] Spalding MD Jarvis GE (2002) The impact of the 1998 coral mortality on reef fish communities in the Seychelles. Mar Pollut Bull 44:309–321. 12139321 10.1016/s0025-326x(01)00281-8

[B36] Tangpu V Yadav AK (2006) Antidiarrhoeal activity of Cymbopogon citratus and its main constituent, citral. Pharmacologyonline 2:290–298.

[B37] Thompson AJ Duke RK Lummis SC (2011 a) Binding sites for bilobalide, diltiazem, ginkgolide, and picrotoxinin at the 5-HT_3_ receptor. Mol Pharmacol 80:183–190. 21505038 10.1124/mol.111.071415 PMC3127528

[B38] Thompson AJ Jarvis GE Duke RK Johnston GA Lummis SC (2011 b) Ginkgolide B and bilobalide block the pore of the 5-HT₃receptor at a location that overlaps the picrotoxin binding site. Neuropharmacology 60:488–495. 21059362 10.1016/j.neuropharm.2010.11.003 PMC3070799

[B39] Thompson AJ Lester HA Lummis SC (2010) The structural basis of function in Cys-loop receptors. Q Rev Biophys 43:449–499. 20849671 10.1017/S0033583510000168

[B40] Thompson AJ Lummis SC (2013) A single channel mutation alters agonist efficacy at 5-HT_3_A and 5-HT_3_AB receptors. Br J Pharmacol 170:391–402. 23822584 10.1111/bph.12287 PMC3834762

[B41] Trattnig SM Harpsøe K Thygesen SB Rahr LM Ahring PK Balle T Jensen AA (2012) Discovery of a novel allosteric modulator of 5-HT_3_ receptors: inhibition and potentiation of Cys-loop receptor signaling through a conserved transmembrane intersubunit site. J Biol Chem 287:25241–25254. 22589534 10.1074/jbc.M112.360370 PMC3408183

[B42] Tuladhar BR Kaisar M Naylor RJ (1997) Evidence for a 5-HT_3_ receptor involvement in the facilitation of peristalsis on mucosal application of 5-HT in the guinea pig isolated ileum. Br J Pharmacol 122:1174–1178. 9401783 10.1038/sj.bjp.0701503 PMC1565058

[B43] Turina AV Nolan MV Zygadlo JA Perillo MA (2006) Natural terpenes: self-assembly and membrane partitioning. Biophys Chem 122:101–113. 16563603 10.1016/j.bpc.2006.02.007

[B44] Walstab J Krüger D Stark T Hofmann T Demir IE Ceyhan GO Feistel B Schemann M Niesler B (2013) Ginger and its pungent constituents non-competitively inhibit activation of human recombinant and native 5-HT_3_ receptors of enteric neurons. Neurogastroenterol Motil 25:439–447, e302. 23490018 10.1111/nmo.12107

[B45] Walstab J Rappold G Niesler B (2010) 5-HT(_3_) receptors: role in disease and target of drugs. Pharmacol Ther 128:146–169. 20621123 10.1016/j.pharmthera.2010.07.001

[B46] Walstab J Wohlfarth C Hovius R Schmitteckert S Röth R Lasitschka F Wink M Bönisch H Niesler B (2014) Natural compounds boldine and menthol are antagonists of human 5-HT_3_ receptors: implications for treating gastrointestinal disorders. Neurogastroenterol Motil 26:810–820. 24708203 10.1111/nmo.12334

[B47] Yip GM Chen ZW Edge CJ Smith EH Dickinson R Hohenester E Townsend RR Fuchs K Sieghart W Evers AS (2013) A propofol binding site on mammalian GABA_A_ receptors identified by photolabeling. Nat Chem Biol 9:715–720. 24056400 10.1038/nchembio.1340 PMC3951778

[B48] Ziemba PM Schreiner BS Flegel C Herbrechter R Stark TD Hofmann T Hatt H Werner M Gisselmann G (2015) Activation and modulation of recombinantly expressed serotonin receptor type 3A by terpenes and pungent substances. Biochem Biophys Res Commun 467:1090–1096. 26456648 10.1016/j.bbrc.2015.09.074

